# Getting Serious about Useful Chemistry Learning: A Case for Attending to Epistemological Messaging

**DOI:** 10.1021/acs.jchemed.5c00829

**Published:** 2025-12-23

**Authors:** Ryan L. Stowe

**Affiliations:** Department of Chemistry, 5228 University of Wisconsin−Madison, Madison, Wisconsin 53706, United States

**Keywords:** Chemistry Education Research, High School/Introductory Chemistry, First-Year Undergraduate, Second-Year Undergraduate, Transfer, Student-Centered Learning

## Abstract

In this Perspective, I consider how our field can take principled actions to align our ways of designing and refining courses with our oft-stated goal for chemistry learning to be useful in daily life. To do so, I make three interrelated arguments. First, I argue achieving this goal will require a particular focus on epistemologies: “[people’s] systems of beliefs [tacit or explicit] about (1) the nature of knowledge and (2) the processes of knowing” [ Educ. Psychol. 2011, 46 (3), 141 ]. Specifically, this goal requires that students (tacitly) experience symmetry between ways of knowing and learning valued in-class and ways of knowing and learning useful in life beyond school. Second, we should compile our sense of useful epistemologies from empirical accounts of people and communities using chemistry to advance personally or professionally meaningful goals: a claim that way of thinking X is useful to dentists should be supported by observations of or interviews with dentists, for example. Third, achieving this goal requires understanding how our course designs communicate allowed epistemologies. Such understandings will enable us to refine our courses such that they better approximate aspects of students’ post-school daily lives.

## Graphical abstract

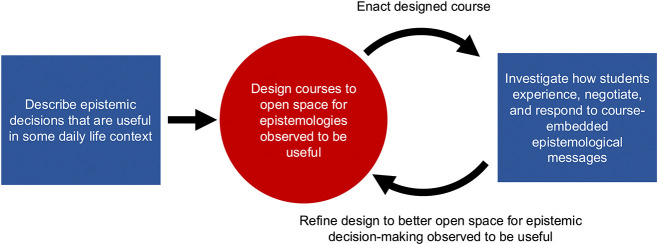

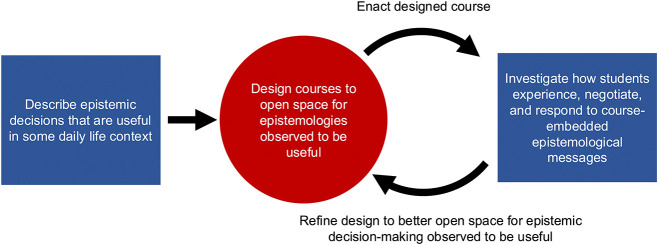

## Introduction

Variations of the following conversation frequently play out in the offices of college chemistry instructors (portions adapted from ref 1):

Students: *I want to be a dentist. Why do I need to take this class?*

Instructor: *Some of the ideas taught in class might help you. For example, we will cover the mechanism by which dental composites harden when you shine blue light on them*.

Student: *That is interesting and all, but I asked my dentist how much they use organic chemistry, and they said, “not at all”. I’m pretty sure you can do a filling without knowing the nuances of how the resin hardens*.

Instructor: *Fair enough, but the ways of thinking you learn in this class will be useful. You will learn skills of pattern recognition, systematic analysis of complex problems, thinking in 3-dimensions, and the application of rules and techniques to new situations. Dentists must surely do these things on a day-to-day basis*.

Student: *Uh huh...*

Here, we see a student questioning the ubiquitous requirement that predental students take organic chemistry. While the instructor initially makes claims about the usefulness of course content, these are quickly deemed implausible by both student and instructor. As a result, the instructor falls back to claiming that the *ways of thinking* emphasized in the course are broadly useful to the profession of dentistry. It is unclear that the student buys what the instructor is selling.

This vignette is meant to make concrete a common strategy in our field. We regularly claim that our courses will help students cultivate ways of constructing and justifying knowledge that are useful in the world outside school.^1–3^ In doing so, we make it seem sensible to require aspiring doctors, marine biologists, dentists, etc. to take our courses. Unfortunately for students like the one in our vignette, claims that ways of knowing and learning useful in-class will also help out in-life are almost entirely rhetorical.^4^ Our field does not build courses around ways of knowing and learning observed to be useful in dentistry (or marine biology, or medicine). Instead, we *a priori* assume there is substantial overlap between how and why students in our classes construct knowledge and how and why dentists construct knowledge. Questions such as the one that began our vignette suggest that, at least some of the time, students are ready to call our bluff.

Empirical work in chemistry and science education supports the concern the student voiced above – that the knowledge and ways of knowing emphasized in-class are not useful in future personal or professional life. Schwarz and colleagues showed that, even in transformed college chemistry classes, students frequently see “the point” of class activities as constructing knowledge products that match the instructor-authored key.^5^ Other ways of justifying knowledge that are more useful in-life (e.g., convincing peers, looking for consistency between claims and what we see) were not experienced as valuable in the focal course. Similarly, Danielak and colleagues tell the story of Michael, who began an engineering program expecting to make sense of phenomena.^6^ Over the course of multiple years, Michael became disenfranchised with how little his classes made space for sensemaking – instead, course assessments overwhelmingly emphasized rote calculations. As a result of experiencing “conceptual things” he valued as peripheral to the main goals of his courses, he considered leaving the program altogether. These two stories make it clear that our classes may be experienced as contrived contexts in which students must quickly construct knowledge that matches instructor expectations. Ways of knowing and learning useful in rapid answer-making are unlikely to be of much use when running a dentist office, exploring a marine ecosystem, or diagnosing a patient.

So how can we take principled actions to align our ways of designing and refining courses with our oft-stated goal that chemistry learning will be useful in daily life?^4^ My answer to this question is 3-fold. First, achieving this goal will require a particular focus on epistemologies - “[people’s] systems of beliefs [tacit or explicit] about (1) the nature of knowledge and (2) the processes of knowing”.^7^ Specifically, this goal requires students (tacitly) experience symmetry between ways of knowing and learning valued in-class and ways of knowing and learning useful in life beyond school. Second, we should compile our sense of useful epistemologies from empirical accounts of people and communities using chemistry to advance personally or professionally meaningful goals – before claiming that “way of thinking X” is useful to dentists, we should talk to/observe dentists, for example. Third, achieving this goal requires understanding how our course designs communicate allowed epistemologies. Such understandings will enable us to refine our courses such that they better approximate aspects of students’ postschool daily lives. In what follows, I elaborate on this answer by drawing on relevant scholarship in science and chemistry education. I am hopeful this contribution helps our community chart a path toward supporting chemistry learning that is useful in later life.

## Our Goals Are Epistemic

Like the instructor in our vignette, we tend to quickly back down from claims that course content will be useful to students outside the classroom. This may be, at least in part, because cursory reflection on our daily activities makes it clear that esoteric chemistry facts and skills are seldom useful. Indeed, this is made light of in commentaries critiquing premedical requirements when the authors ask questions like: “do any physicians, even researchers, have to know about Diels-Alder adducts?”^1^ The implicit answer is “no, of course not”.

When faced with the potential uselessness of course content, we often pivot to making claims about the ways of thinking needed to engage with this content. This too was previewed by our vignette. Specifically, we hope that our classes will help students “make informed decisions, build justifications, evaluate outcomes, and test ideas in the context of personal, societal, and global concerns.”^8^ These goals are about ways of constructing and justifying knowledge that advance personally meaningful aims – that is, they are about learners’ epistemologies.

Scholarship in science education teaches us that a focus on epistemology is sensible if our goal is supporting daily life-decision-making.^9,10^ Specifically, work by Engle showed that transfer occurs when students see themselves as knowers in a community that is temporally connected to contexts in which they will use their knowledge.^10^ Hammer and colleagues further elaborate on the mechanisms by which epistemology may drive these local temporal connections. They theorize that, if one’s expectations for “what is going on here”^11^ are sufficiently similar in two scenarios, then one is likely to transfer knowledge and ways of knowing deemed useful in one scenario to the other.^9^ This means that, if we want to support students in reasoning with and about chemistry in-life, we need to develop and refine courses that approximate daily life contexts. We want students in their later lives to (perhaps tacitly) think, “this moment of epistemic decision-making seems similar to something I did in chemistry class. I bet the ways I learned to construct and justify knowledge there will also help me here.”

Advocating we focus especial attention on epistemology may seem inappropriate to some. After all, one can parse useful chemistry learning in other ways (e.g., content useful in daily life, affective states useful in daily life) so why elevate epistemology? Here, I follow the work of other scholars^12–18^ to argue that other ways of parametrizing “useful in daily life” flow from or into epistemology. For example, the type of content knowledge students bring to bear in class activities is bounded by their epistemic understanding of those activities. We see evidence of this in Lising and Elby’s description of Jan^13^ who, as a consequence of adopting an epistemology in her physics course that only valued formal knowledge, failed “to use the skills or knowledge [she] clearly possess­[ed]” (p. 381). As such, altering what knowledge matters in a chemistry course will involve shifting students’ epistemic understandings of school science. Relatedly, epistemological understandings of what and whose knowledge “counts” in our classes affects who is positioned as a legitimate knower and who is not.^16,17^ This matters because using chemistry in later life will require one see oneself as capable of thinking about and with molecular-level ideas (i.e., see oneself as a chemistry knower). Undermining students’ status as chemistry knowers thus has implications for engaging with chemistry in life beyond school.^17^

### How We Should Model Epistemology

Considering how we might open space for students to experience symmetry between epistemologies useful in-class and those useful in-life requires us to be specific about the “kind of thing” epistemologies are. Thankfully, many decades of research in science education can help us.^9,19–21^ Here, I draw on empirical work done in K-16 STEM courses that shows students’ epistemologies to be dynamic, highly variable, and context-sensitive.^9,20,22^ To account for these observations, I adopt a model of epistemology as made up of many fine-grained, multidimensional “resources”^20^ or “pieces”^23^ that individuals bring together in local moments of meaning-making. This model of epistemologies is consistent with recent work around epistemic cognition.^19^ I refer to epistemologies (rather than epistemology) as a way to remind readers of this theoretical stance toward the ontology of epistemology.

There are a few key aspects of this model. First, unlike related theories of the nature of science,^24–26^ epistemological resources are neither right nor wrong. For example, the idea that knowledge is learned from authority is neither correct nor incorrect. Instead, we can use the idea that knowledge is learned from authority productively in some contexts (when my doctor recommends a certain medication) and not in others (when arguing with my partner about what color paint to use on our house).

The second aspect of the epistemological resources model that is important for my work here is that epistemologies are situated in context—with different situations calling for different epistemologies.^27,28^ This feature of epistemologies necessitates that we have a suite of resources that we can look through and draw from when encountering the wide range of situations in which we need to construct knowledge on a daily basis. For example, we need to have a “knowledge from authority” resource when talking to our doctors but also something like “knowledge is constructed” when arguing with our partners.

A third feature of epistemologies key to my work here is their multidimensionality. Much of the literature on epistemologies has sought to identify the various aspects of knowledge and knowing that epistemological knowledge may address. For example, early on Hofer and Pintrich identified three “consensus” dimensions of epistemology including: Certainty vs Tentativeness, Authority vs Independence, and Simplicity vs Complexity.^29^ Since that time, researchers have expanded and refined dimensions of epistemology relevant to student science learning. In this article, I will use the language of Chinn and colleagues’ AIR model of epistemic cognition^30^ to consider goals animating knowledge construction (epistemic aims), the relative worth of these goals (epistemic values), criteria by which we know our goals have been achieved (epistemic ideals), and knowledge construction processes useful for goal attainment (reliable processes). I do so because this model encompasses insights from many scholars in philosophy and science education.

Taken together, these three features of epistemology mean that we cannot define epistemological development as progressively moving toward a single “more correct” epistemology. Instead, there are a range of ideas about knowledge and knowing that are more and less valid and useful in different real-life contexts. It is thus not appropriate for me (or anyone else) to define “better” or “worse” epistemologies *a priori*. Instead, epistemological development involves possessing and being able to access a diverse set of epistemological resources to achieve worthwhile epistemic aims.^12,31,32^

If we model epistemic cognition as context-dependent resource activation, we must sharpen how we define our goals. Specifically, we must move beyond stating that students should “make informed decisions, build justifications, evaluate outcomes, and test ideas in the context of personal, societal, and global concerns”^8^ toward considering which contexts matter and what sort of epistemic decisions are useful in navigating these contexts. I agree with Feinstein that answers to questions about important contexts and useful epistemologies ought not to be made deductively by a room full of chemistry instructors.^4^ We do not, as it turns out, generally have a nuanced understanding of epistemologies useful in navigating dentistry, emergency medicine, civic action against corporate polluters, etc. As such, our claims about what sort of epistemic decision-making is useful in what contexts should be informed by empirical work detailing how people and communities use science as they grapple with practical concerns and interests.

In the next section, I illustrate how one might use a sociological account of people reasoning with and about chemistry to construct epistemic goal spaces useful for informing course design. In doing so, I am not suggesting the contexts described by this account are the only or most important contexts one might consider. Instead, I wish to illustrate the kind of inferences about useful epistemologies that can be informed by empirical accounts of people’s and community’s reasoning. It would be generative for our field to be in conversation with our students, their communities, and colleagues in Science & Technology studies (STS) about other contexts that might be worth attending to as we think about supporting useful chemistry learning.

## Chemistry for Civic Action

Sociological accounts of people reasoning with and about science can teach us about the nature and impact of epistemic decisions that are useful in daily life. We can learn about the sort of goals that animate knowledge construction in-life and how (or whether) people had a say in choosing these goals. Likewise, we can see what criteria were used to know goals were achieved and how these criteria were negotiated in a given situation. We might also get evidence of (un)­reliable processes used to work toward aims that mattered to people and communities. Importantly, sociological accounts should **not** be read as suggesting some collection of “best” epistemologies. There will be idiosyncratic aspects of all scenarios STS scholars explore, and these will affect which epistemic ideas seemed useful when. Instead, our task is to identify a space of commonly encountered epistemic decisions in order that we might engage students in our classes in making similar decisions. Below, I take a “deep dive” into one account of people using chemistry in-life and, from this account, suggest aspects of epistemic decision-making that were useful in our focal scenario. Specifically, guided by Anna Clark’s book length account,^33^ I explore how residents of Flint, Michigan engaged with science to stem the tide of toxic water flowing into their homes and bodies. I center sociopolitical action in what follows (instead of dentistry) for two reasons: 1) there exist many rich sociological accounts of people using science for civic action;^33,34^ I am unaware of analogous accounts describing dentists’ epistemic decision-making, and 2) most of the students in our courses will not go on to be dentists. That said, if we wish to claim that predental students should take chemistry because they will learn useful ways of thinking, we should carry out empirical work to examine (mis)­alignment between epistemologies useful in-dentistry and epistemologies encouraged by our classes.

### The Case of the Flint Water Crisis

Before 2014, Flint’s water was supplied by the Detroit Water and Sewer Department (DWSD). The DWSD drew water from nearby Lake Huron, treated this water in a plant near the lakeshore, and pumped treated water to the city of Flint. This water was both safe and expensive – Flint residents paid more for water than residents of most neighboring towns. As such, when considering how to manage Flint’s substantial deficit, cutting water costs seemed an attractive option.^33^

To bring down the cost of water in Flint, a plan was devised to supply the city with raw water from Lake Huron. This water would be treated by Flint’s ancient water treatment plant and then distributed throughout the city. Unfortunately, this plan required new infrastructure and, while this infrastructure was being built, it was decided the city would draw water from the nearby Flint river and treat this water locally.^33^ This plan encountered some opposition - Flint’s water administrator at the time noted that the local treatment plant was not ready to treat raw water from the Flint River.^35^ Unfortunately, objections such as this were ignored, and Flint began distributing river water to residents in the spring of 2014.

Soon after the water switchover, Flint residents began noticing that their water smelled and tasted off. They were right to worry. Due to the local water treatment plant not adding corrosion control, lead and iron were dissolved in Flint’s water at dangerously high levels. Lead accumulates in teeth, bone, and soft tissues and causes a wide range of issues (e.g., kidney failure, reduced attention spans, seizures, coma). Harms of lead poisoning are especially acute in children. This means that changes in Flint’s water supply caused substantial and lasting harm to the people of Flint, especially children who lived, worked, and played in and around the city.^33^

Tragically for the citizens of Flint, the 2014 water crisis can be read as a struggle against authorities who were inclined to dismiss the testimony and evidence of Flint residents and collaborating scientists. The findings of a city-wide water quality study, in which >250 samples were collected by community members and analyzed by Virgina Tech chemist Marc Edwards’ team, were dismissed by the Michigan Department of Environmental Quality (MDEQ). Edwards’ team was described by a department spokesperson as outsiders who had “just arrived in town and quickly proven the theory they set out to prove”.^33^ Mona Hanna-Attisha’s analysis of blood samples collected from Flint’s children – which showed the lead concentration in blood nearly doubling 18 months after the water switchover – was likewise initially dismissed. Mona was accused of “near hysteria” and a communication official with the Michigan Department of Health and Human Services (MDHHS) said that this surge in blood-lead levels was “seasonal and not related to the water supply”.^36^ Ultimately, *The Detroit Free Press* reanalyzed data on lead in the blood of Flint children and demonstrated that the MDHHS had misinterpreted their own findings.^36^ This led to a cascade of actions that resulted in Flint switching their water supply back to the DWSD.

### Epistemic Decisions Useful in Flint

Flint residents made many epistemic decisions as they fought for safe water. Below, we describe the substance of some of these decisions – that is, whether they pertained to epistemic aims, ideals, or reliable processes.

#### Epistemic Aims

Flint residents had epistemic aims of various grain sizes. Perhaps the largest grain of these was an argument that prompted authorities (e.g., MDHHS, MDEQ) to take substantive action to improve water quality in their city. Achieving this goal necessitated working toward many smaller-grain aims such as an understanding of sound water sampling procedures and an epistemic network^37^ that included credible scientists. Small grain aims were useful if achieving these brought citizens’ concerns to the attention of officials who could affect Flint’s water and spurred these officials to act.

The ways in which achievement of smaller-grain aims aggregated to lead to something larger were not linear or predictable. For example, Flint resident LeeAnn Walters carefully collected 30 water samples from her house and sent these to Miguel Del Toro, a regional groundwater regulations manager for the Environmental Protection Agency (EPA). Her aim in doing so was to generate evidence that her water was toxic and required remediation. She received this evidence in the form of a report Del Toro entitled “High Lead Levels in Flint, Michigan”. This report summarized the situation in Flint as follows: “I understand that this is not a comfortable situation, but the State is complicit in this and the public has a right to know what they are doing because it is their children that are being harmed”.^33^ LeeAnn shared Del Toro’s draft report with several journalists she trusted. Here, her aim was to raise the profile of report findings to spur some sort of useful action by the state or city. In this, LeeAnn achieved some success. Disseminating Del Toro’s report helped inspire the short film *Hard to Swallow: Toxic Water under a Toxic System in Flint* as well as interviews with state and local officials on Michigan Public Radio.^33^

#### Epistemic Ideals

Criteria used by Flint residents to judge aim achievement were different for different aims. For example, Flint residents would know their largest-grain aim was achieved when their city’s water supply switched back to Lake Huron. Smaller grain aims (e.g., understanding trustworthy water sampling protocols) had different criteria, such as plausibility to relevant experts. Consistency with lived experience was a particularly salient epistemic ideal for many epistemic aims pursued by Flint residents. Many residents, including LeeAnn Walters, were spurred to action due to inconsistency between their lived experiences and the claims made by state and local officials.

#### Reliable Processes

Epistemic processes are reliable if they enable progress toward an epistemic aim.^30^ Reliable processes evident in Flint residents’ activism included persistently contesting authorized accounts of water quality, assembling a city-wide coalition to sample water at hundreds of sites, and distributing the results of water and blood tests to investigative journalists.

Now that we have briefly unpacked the substance of some of the epistemic decisions made by the people of Flint, we turn to considering how (or whether) Flint residents could make choices about salient aims, ideals, or reliable processes. Flint residents had little say over the large-grain aim that animated their engagement with scientific ideas and practices. They were being poisoned by their city’s infrastructure and had to do something, or they would continue to suffer harm from lead in the water. Other communities who have been similarly affected by toxic surroundings were likewise forced into action.^34^ Smaller grain aims, like justified arguments, or connections to useful sources of knowledge, could be shaped by residents’ choices. Criteria for achieving resident’s large-grain aim of prompting infrastructural changes emerged through dialogue with city officials and scientist collaborators. There were no prespecified criteria that guaranteed residents’ arguments, explanations, etc. would be taken seriously. Activists in Flint had substantial latitude to make choices about epistemic processes that might enable achievement of aims that prompted civic action. These included making connections with scientific authorities and regulators, building coalitions of residents in order to aggregate lived experiences, and directly confronting officials with evidence of harm. There was no way to know *a priori* whether these processes would achieve desired smaller-grain epistemic aims, nor whether aggregation of smaller-grain aims would lead to the widespread change sought by the people of Flint. As such, the capacity to flexibly adjust epistemic aims, ideals, and reliable processes was important to Flint residents’ activism.

## Supporting Civic Action in Chemistry Class: A Role for Epistemological Messaging

Suppose we want to equip our students to engage in activism like what unfolded in Flint during the water crisis of the mid-2000s. To do so, we would need to first understand the substance and impact of decisions about knowing and learning that were available and useful for achieving residents’ aim of prompting civic action (described above). We would then need to theorize about how course design and enactment might open space for students to make epistemic decisions similar to those engaged in by members of the Flint community. That is, students would need to experience symmetry between epistemologies useful in-class and those that might be useful in civic action. Work by Russ can support this sort of theorizing.^32^ She argues that messages about valued knowledge and ways of knowing (i.e., epistemological messages) are tacitly embedded in teachers’ talk during instruction. Subsequent work extended the construct of epistemological messaging by demonstrating that such messages are also embedded in other facets of a course ecosystem, such as assessments and related answer keys.^5^ Epistemological messages matter because students’ experiences with these messages can shape how, why, and what they learn. For example, Rosenberg and colleagues observe a student group shift from accumulating facts from worksheets toward making sense of a phenomenon after they experience an epistemological message from their teacher.^22^ With this work in mind, we might more precisely describe our task of approximating Flint-like contexts in-class as embedding epistemological messages in our course designs that we think might open space for students to negotiate aims, ideals, and reliable processes in ways that mirror Flint residents’ decision-making. These designs would “work” if we see students engage in the sorts of epistemic decision-making we were hoping to support.

To more concretely illustrate what epistemological messaging affords design projects intent on supporting epistemological learning useful in daily life, I sketch a design-based research (DBR) project that centers a subset of epistemologies observed to be useful in Flint. To do so, I make use of Sandoval’s conjecture mapping approach, shown in generic form in Figure 1 below.

**Figure 1:**
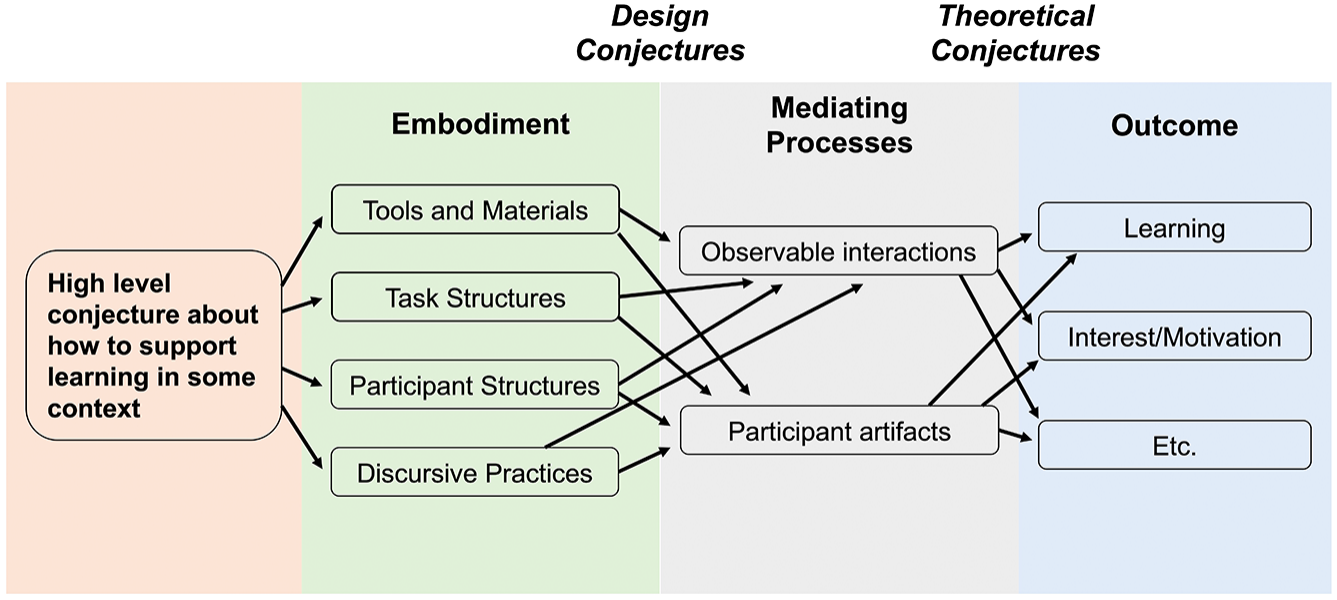
Generalized conjecture map describing a design-based research program. Adapted from ref 38 with permission from Taylor & Francis, Ltd.

Conjecture maps of the sort shown in Figure 1 specify six major elements and how they are related. Designs begin with one or more *high-level conjectures* about the kind of learning designers hope to support. These conjectures are *embodied* by designed tools, participant structures, discursive strategies, etc. If designed materials are enacted in certain ways (*mediating processes*) then researchers would expect to see desired *outcomes. Design conjectures*, which are useful for refining embodiments (*e.g*., curricula, activity structures), are ideas about how designed materials would be expected to generate mediating processes. *Theoretical conjectures*, which are useful for advancing theories of learning, specify how mediating processes produce desired outcomes. Note that mediating processes are central to refinement of both designs and related theory. DBR requires that we open the “black box”^39^ of the learning environment to explore linkages between concrete realizations of a design, use of these embodiments in naturalistic settings, and outcomes of interest.

My hypothetical DBR project is built around the high-level conjecture that *prompting sociopolitical action requires broadening who those with authority consider a knower*. Throughout the Flint water crisis, resident activists were routinely positioned as nonknowers for the purpose of dismissing their testimony. My high-level conjecture is meant to crystallize why members of the Flint community formed connections and eventually collaborated with members of the scientific community (e.g., Marc Edwards) – they recognized (perhaps tacitly) that science is a language of power and that knowing scientists and engaging in scientific work might afford access to this language and the authority it can grant.^40,41^ Stated using the language of AIR, achieving large-grain epistemic aims that spur sociopolitical action requires that activists be seen as legitimate knowers by those with authority.

Embodiments, including curricular materials and task + participant structures, would be designed to open space for students to consider what role science could (or should) play in legitimizing the lived experiences of communities experiencing harm from environmental pollution. For these conversations to generate learning useful beyond the classroom, students would need to connect their conversations to “matters of consequentiality – sociopolitical and socio-economic concerns facing and impacting students’ communities, pasts, presents, and futurities”.^42^ To encourage such connections, I would center my unit on a locally relevant instance of environmental injustice: the aluminum die casting factory Madison – Kipp introducing toxins into the air, water, and soil of the Atwood neighborhood in Madison, WI.^43^ Unit activities would open space for students to explore ways in which industrial actors pollute the environment around campus and consider what should be done to curtail and remediate this pollution. Some activities would involve chemistry ideas and practices (e.g., quantifying toxin concentrations in water and soil and how these change across time) while others would focus on ideas and practices that typically fall outside school chemistry (e.g., considering how the glacial pace of toxic substance regulation serves industrial interests^44^). To add nuance to students’ understandings of environmental injustice in their backyard, the class would connect with local activists who played a role in holding Madison – Kipp accountable for polluting Madison neighborhoods.^45^ Conversations with these activists, as well as students’ analysis of environmental contaminants and understandings of relevant regulations, would inform construction of strategies for prompting action from state and local authorities. The unit would conclude by the class receiving feedback on these strategies from officials who work in the Wisconsin Department of Natural Resources. This feedback would help the class calibrate the characteristics of epistemic aims that are likely to move the needle on sociopolitical change (i.e., epistemic ideals).

Successfully realizing the narrative sketched above would require carefully embedding epistemological messages throughout designed materials and activity structures. Students would need to, at various times, experience their role as constructing evidence-based arguments about environmental contamination in their city, explaining how regulations might open or close space for industrial actions, interpreting activists’ testimonies, and coming up with possible plans to advance environmental justice in Madison and beyond. To help make this happen, I would theorize about curricular structures (e.g., prompting strategies) and ways of enacting these structures that might communicate the utility of epistemologies I hope to surface. For example, after the class analyzes and interprets data on chemical contaminants around campus, I may open space for consideration of ways in which scientific evidence has contributed to sociopolitical change. Specifically, in-class conversations and related readings^34^ would be designed/curated to communicate that scientific findings often have a limited role in shifting industry behavior or regulations that might constrain that behavior. This realization would motivate the class to investigate characteristics of successful activist efforts – both how these unfolded and what coalition of actors was needed to prompt change. I expect these activities would communicate that civic action is often advanced by building connections between parties with different knowledge, expertise, and authority – curating epistemic networks^37^ is thus one of the reliable processes commonly seen in successful instances of civic action.

Inferring whether my design works (or not) would be about exploring whether students experience design-embedded epistemological messages in the ways I expect. I can investigate this via inferring different epistemological ways of being from behavior^15^ or via analyzing dialogue from carefully structured interviews that invite students to reflect on how their sense of “what is going on” in class was influenced by particular design-embedded epistemological messages.^5^ Both types of investigation are challenging both analytically and theoretically. I would need to, for example, figure out theoretically coherent ways of deciding which class moments are worth attending to and how messages from class and elsewhere aggregate for particular students/student groups across the arc of instruction. Conducting this sort of work in a way that can inform quick, iterative cycles of DBR would be a substantial hurdle. However, I believe all of these challenges are surmountable by thoughtful scholars in our field working in partnership with learners and their communities. It is possible to attend to shifts in epistemological resource activation over a long time scale,^46^ and to consider how features of a learning environment communicate valued ways of knowing and learning over the course of days to weeks.^5,47^

My outcomes would be achieved (or not) if I regularly see evidence of class engagement in epistemic decision-making that is similar to what we saw in Flint. If class-embedded epistemological messages were not experienced as intended, then I would potentially adjust my high-level conjecture (*e.g*., by further specifying whose experiences most need legitimizing and how this might be done) and/or how such conjectures are embodied (*e.g*., by changing the ways in which a given message is expressed in materials or task + participant structures).

In sketching this hypothetical design project, I attempted to illustrate how sociological accounts of epistemic decision-making might inform the epistemological messages we embed in the courses we design and enact. To streamline my argument, I neglected many practical concerns that might stand in the way of focusing chemistry class on epistemologies useful for civic action. For example, I made no assumptions about course content expectations, nor how (or whether) engagement in my designed unit would prepare students for subsequent courses. In this Perspective, I cannot adequately attend to the many challenges that might arise if creating a new course or integrating a civic chemistry unit into an existing course. I can, however, note that our colleagues in science education have successfully opened space for Advanced Placement chemistry students to grapple with locally relevant social justice science issues.^48–50^ This work demonstrates that it is possible and productive to center goals other than content understandings in our chemistry courses.

## Concluding Thoughts

In this Perspective, I have worked to add nuance to our goal of supporting chemistry learning that is useful in daily life. Specifically, I have argued that this goal is about cultivating ways of knowing and learning (i.e., epistemologies) with post school utility. I then joined Feinstein^4^ in advocating that our field calibrate what epistemologies are useful when by engaging with empirical accounts of people using science to advance practical concerns and interests. I illustrated what this might look like by exploring epistemic decision-making that was useful as Flint residents fought for clean water in their city. Finally, I argued that supporting useful epistemologies in-class requires a way to connect course design and enactment to how, why, and what students learn in that class. We can do so by intentionally embedding epistemological messages^32^ into designed materials and investigating ways in which students experience, negotiate, and respond to these messages. Course designs can then be refined to increasingly align students’ experiences with course-embedded messages with our goals.

A great deal more could be written about each of the three arguments threaded through this piece. My goal here is not to be comprehensive. Instead, I hope to template conversations our field might engage in as we work to carefully define and defend our goal spaces and create classes that support these goals. I look forward to dialogue with chemistry educators and the broader science education community about how we might undertake this theoretically and analytically challenging work.
